# Phenylalanine transfer across the isolated perfused human placenta: an experimental and modeling investigation

**DOI:** 10.1152/ajpregu.00405.2015

**Published:** 2015-12-16

**Authors:** E. M. Lofthouse, S. Perazzolo, S. Brooks, I. P. Crocker, J. D. Glazier, E. D. Johnstone, N. Panitchob, C. P. Sibley, K. L. Widdows, B. G. Sengers, R. M. Lewis

**Affiliations:** ^1^Faculty of Medicine, University of Southampton, Southampton, United Kingdom;; ^2^Bioengineering Science Research Group, Faculty of Engineering and the Environment, University of Southampton, Southampton, United Kingdom;; ^3^Maternal and Fetal Health Research Centre, Institute of Human Development, University of Manchester, and St. Mary's Hospital and Central Manchester University Hospitals NHS Foundation Trust, Manchester Academic Health Science Centre, Manchester, United Kingdom; and; ^4^Institute for Life Sciences, University of Southampton, Southampton, United Kingdom

**Keywords:** blood flow, amino acid transfer, exchanger, facilitated transport, metabolism

## Abstract

Membrane transporters are considered essential for placental amino acid transfer, but the contribution of other factors, such as blood flow and metabolism, is poorly defined. In this study we combine experimental and modeling approaches to understand the determinants of [^14^C]phenylalanine transfer across the isolated perfused human placenta. Transfer of [^14^C]phenylalanine across the isolated perfused human placenta was determined at different maternal and fetal flow rates. Maternal flow rate was set at 10, 14, and 18 ml/min for 1 h each. At each maternal flow rate, fetal flow rates were set at 3, 6, and 9 ml/min for 20 min each. Appearance of [^14^C]phenylalanine was measured in the maternal and fetal venous exudates. Computational modeling of phenylalanine transfer was undertaken to allow comparison of the experimental data with predicted phenylalanine uptake and transfer under different initial assumptions. Placental uptake (mol/min) of [^14^C]phenylalanine increased with maternal, but not fetal, flow. Delivery (mol/min) of [^14^C]phenylalanine to the fetal circulation was not associated with fetal or maternal flow. The absence of a relationship between placental phenylalanine uptake and net flux of phenylalanine to the fetal circulation suggests that factors other than flow or transporter-mediated uptake are important determinants of phenylalanine transfer. These observations could be explained by tight regulation of free amino acid levels within the placenta or properties of the facilitated transporters mediating phenylalanine transport. We suggest that amino acid metabolism, primarily incorporation into protein, is controlling free amino acid levels and, thus, placental transfer.

understanding the determinants of placental function and fetal growth are important, as poor fetal growth is associated with impaired health throughout life (14). Amino acid transfer, a key placental function required for fetal growth, is reduced in growth-restricted pregnancies (23). To understand why placental amino acid transfer becomes restricted in these pregnancies, we need to define the factors that may be limiting to this process. It is clear that net placental amino acid flux to the fetus is dependent on membrane transport proteins localized to the microvillous membrane (MVM) and basal plasma membrane (BM) of the syncytiotrophoblast (9). However, other variables, such as blood flow and metabolism, could be equally limiting to net placental amino acid transfer (18).

In growth-restricted pregnancies, umbilical blood flow may be reduced by 50%, impairing transfer of oxygen and, potentially, other nutrients to the fetus (1). Substances predominantly transferred by diffusion, such as small hydrophobic solutes, are most likely to be sensitive to flow, as their net flux is driven by concentration gradients maintained by maternal and fetal blood flows. Under these circumstances, maintenance of a transplacental concentration gradient is a key determinant of oxygen transfer by simple diffusion and glucose transfer by facilitated diffusion (5, 10, 29). For substances predominantly transferred by active transport (charged and/or hydrophilic solutes), maternal blood flow is necessary to deliver substrates for transfer to the transporting plasma membrane, but flow is less likely to be the rate-limiting step, as transfer is not directly dependent on transplacental concentration gradients.

Amino acid transfer across the placenta is an active process that occurs against a concentration gradient (6, 9). As such, placental amino acid transfer has not generally been considered to be flow-limited. Nevertheless, many of the amino acid transporters involved in this process do rely on transmembrane concentration gradients (8, 22). In particular, transfer of amino acids from the placenta to the fetal circulation, across the BM, is mediated by facilitated transporters and exchangers, both of which rely on transmembrane amino acid concentration gradients across the plasma membrane for their activity (7, 8). With fetoplacental blood flow determining amino acid concentrations in the fetal capillaries, the issue of flow dependency of amino acid transfer from the placenta to the fetus is raised in the context of presiding concentration gradients across the BM. As amino acid concentrations are believed to be much higher within placental tissue than in fetal capillaries, any change in transmembrane concentration gradient due to flow is likely to be relatively small (24). Hence, the effect of fetal flow on transfer would be predicted to be small but requires experimental validation.

Flow-limited transfer has been studied previously in the isolated perfused human placenta and has been clearly established, as expected, for antipyrine (28). There is also evidence that maternal flow rate affects transfer of glucose across the isolated perfused human placenta (15). Modeling of placental amino acid transfer also suggests that flow may be an important determinant (18). Phenylalanine is a good candidate amino acid with which to study possible flow effects, as it is transported by exchangers [SLC7A5 (LAT1) and/or SLC7A8 (LAT2)] and facilitated transporters [SLC16A10 (TAT1), SLC43A1 (LAT3), and SLC43A2 (LAT4)], the activity of which is dependent on concentration gradients that are sensitive to flow (8, 22). Phenylalanine, taken up by the placenta, may be incorporated into protein; however, as there is little or no phenylalanine hydroxylase in the human placenta, loss via catabolism is likely to be limited (20). Using experimental and modeling approaches, we set out to investigate whether factors such as maternal and fetal blood flow influence placental transfer of the amino acid phenylalanine as a model for essential amino acid transport by exchangers and facilitated transporters across the human placenta.

## METHODS

Human placentas were collected from daytime full-term vaginal deliveries from uncomplicated pregnancies at the Princess Anne Hospital in Southampton, in accordance with ethical approval from the Southampton and Southwest Hampshire Regional Ethics Committee (approval no. 11/sc/0323).

### 

#### Perfusion methodology.

Placentas were perfused using the methodology of Schneider et al. (28), as adapted in our laboratory (10, 11). Placentas were collected within 30 min of delivery and placed on ice for transport to the laboratory, where fetal-side perfusion was established within 30 min of collection. The fetal and maternal circulations were perfused at 6 and 14 ml/min, respectively, with Earle's bicarbonate buffer (EBB; in mM: 1.8 CaCl_2_, 0.4 MgSO_4_, 116.4 NaCl, 5.4 KCl, 26.2 NaHCO_3_, 0.9 NaH_2_PO_4_, and 5.5 glucose) containing 0.1% (wt/vol) bovine serum albumin and 5,000 IU/l heparin and equilibrated with 95% O_2_-5% CO_2_. Perfusion of the fetal circulation was established, and, if fetal venous outflow was ≥95% of fetal arterial inflow, maternal arterialerfusion was established 15 min later. Maternal arterial catheters are placed through the maternal decidual surface of the placenta and into the intervillous space. The maternal venous outflows are not catheterized, but maternal venous exudate appearing on the surface of the cotyledon was channeled to a collection point.

#### Phenylalanine experiment methodology.

**Fig. 1.**
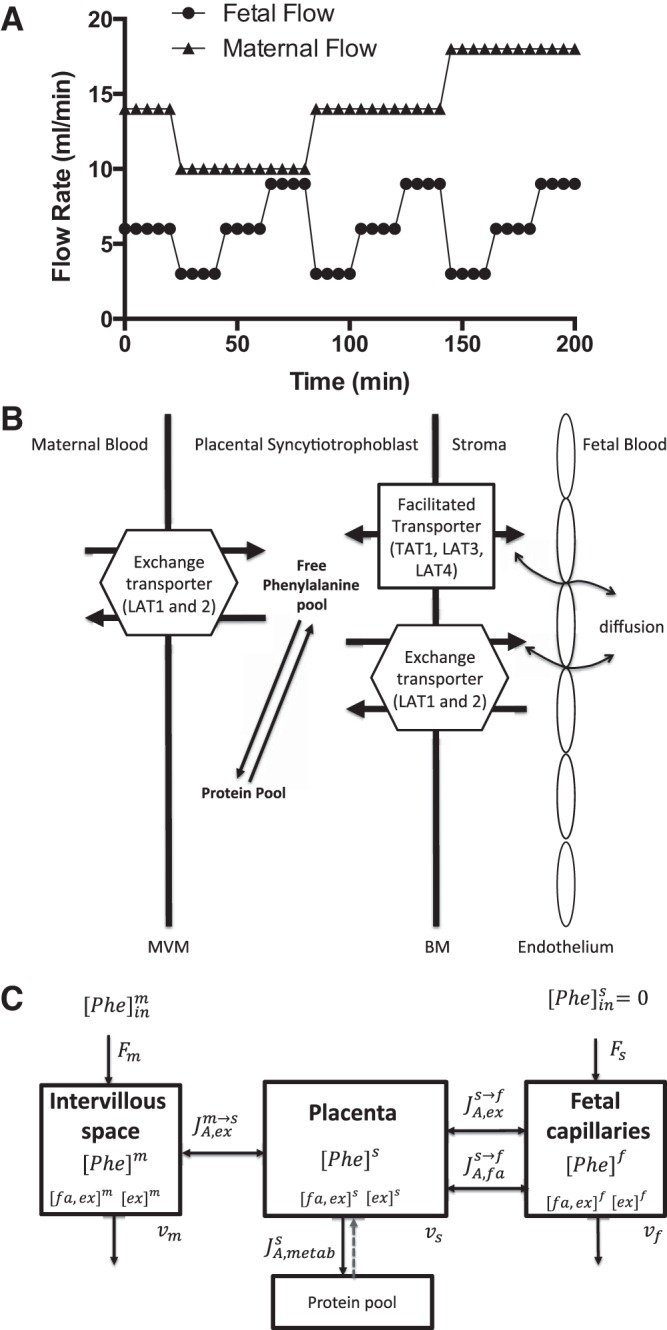
Experimental design and modeling schematic. *A*: experimental design. Stepwise changes in maternal and fetal perfusion flow rates from the beginning of [^14^C]phenylalanine tracer infusion. After an initial 20-min equilibration period, flow rates were varied every 20 min and maternal and fetal venous outflow samples were collected at 15 and 18 min to determine uptake and transfer, respectively. *B*: conceptual outline of phenylalanine transport across the human placenta showing the classes of transporters involved on the microvillous (MVM) and basal (BM) plasma membranes of the placental syncytiotrophoblast, as well as incorporation into the protein pool (catabolism is not shown, as phenylalanine hydroxylase is not expressed in the placenta). *C*: compartmental computational modeling of transporter-mediated phenylalanine transfer. F is flow in maternal or fetal arteries, [Phe] is [^14^C]phenylalanine concentration in the respective compartments of the maternal intervillous space (m), syncytiotrophoblast (s), and fetal capillaries (f), and v is compartment volume. *J* represents net flux between compartments for exchangers (ex) or facilitated transporters (fa), and metabolism is given by *J*_metab_. While it was assumed that metabolism of phenylalanine was predominantly protein synthesis, which is reversible, given the short experimental time frame, flux of [^14^C]phenylalanine back from protein to the free amino acid pool was not modeled (dashed arrow). Phenylalanine uptake in the placenta via exchange is driven by high intracellular concentrations of endogenous substrates of the facilitated and exchange (fa and ex) or exchange-only (ex) transporters.

Phenylalanine was chosen as the candidate amino acid, as it is a substrate for both exchangers and facilitated transporters (8). It is also not catabolized (i.e., phenylalanine hydroxylase is not expressed) within the human placenta (20). Tracer concentrations of phenylalanine were used to study flow in an experimental design where transporters were not saturated to ensure that any effects of flow were apparent. Glutamate and taurine were added to support metabolic and tissue homeostasis within the perfused placental tissue (11, 12). The maternal circulation was perfused with EBB containing 2.7 nmol/l [^14^C]phenylalanine [50 μCi (1.85 Mbq), NEC284E050UC], 50 μmol/l glutamate, and 50 μmol/l taurine, along with 1.8 mmol/l creatinine as a marker of paracellular diffusion. Initial baseline maternal and fetal flow rates were 14 and 6 ml/min, respectively, for 30 min. As outlined in Fig. 1, maternal flow rate was then changed from 14 ml/min to 10 ml/min back to 14 ml/min and then to 18 ml/min for 1 h each. During each 1-h period, fetal flow rates were ramped to 3, 6, and 9 ml/min for 20 min each. In each 20-min block, maternal and fetal venous exudates were sampled at 5, 10, 15, and 18 min. Finally, the tissue was washed by perfusion of both circulations for 15 min with buffer that did not contain [^14^C]phenylalanine. After the perfusion protocol, the cotyledon was trimmed of nonperfused areas (perfused areas become blanched), and the cotyledon was frozen for analysis of intracellular amino acids.

Placental uptake (mol/min) was calculated from the difference in concentration (mol/l) between maternal arterial and maternal venous outflow, multiplied by maternal flow rate (l/min). Placental transfer (mol/min) was calculated from fetal vein concentrations (mol/l) multiplied by fetal flow rate (l/min).

#### Tissue amino acid measurements.

To study intracellular [^14^C]phenylalanine, the cotyledons were homogenized in three volumes of distilled water and centrifuged at 10,000 *g* for 10 min to remove cellular debris. A 1-ml sample of homogenate was mixed with an equal volume of 10% trichloroacetic acid to precipitate protein. The pellet and supernatant were counted separately in a liquid scintillation counter (Tri-Carb 2100TR, Perkin Elmer Life Sciences) on standard counting windows for ^14^C to determine [^14^C]phenylalanine incorporated into protein and free ^14^C tracer. To assess potential quenching of radioactive counts by tissue components, the supernatant and protein pellet were prepared as described above, and serial double dilutions were performed, with each sample being spiked with a standard amount of ^14^C tracer and counted as described above. No quenching was observed in the supernatant, but within the protein pellet, efficiency of counting was 31%, and these counts were adjusted accordingly.

#### Computational modeling.

A compartmental model to represent the intervillous space, syncytiotrophoblast, and fetal capillaries was constructed using relative volume fractions from the literature (30). Flow rates were based on the experimental protocol outlined in Fig. 1, and overall cotyledon volume was based on the average value from these experiments (with the assumption of 1 ml/g tissue). The placenta was modeled with generic exchange and facilitated transporters as outlined in Fig. 1, *B* and *C*. Model equations were implemented in MATLAB (R2014b) as outlined previously (22, 30).

#### Transport modeling.

A carrier-based model was used to represent the transporters, as outlined previously (22, 32). Net flux *J*_*A*_^*I*→*II*^ (mol/min) of *substrate A* from *compartment I* to *compartment II* for the exchanger (ex) model is given by

$$J_{A,ex}^{I\to II}=V_{ex}\frac{{\left[A\right]}^{j}{\left[R\right]}_{ex}^{II}-{\left[A\right]}^{II}{\left[R\right]}_{ex}^{L}}{K_{ex}\left({\left[Tot\right]}_{ex}^{I}+{\left[Tot\right]}_{ex}^{II}\right)/2+{\left[Tot\right]}_{Bx}^{I}{\left[Tot\right]}_{Bx}^{II}}$$

and by

$$J_{A,fa}^{I\to II}=V_{fa}\left(\frac{{\left[A\right]}^{I}}{K_{fa}+{\left[Tot\right]}_{fa}^{I}}-\frac{{\left[A\right]}^{II}}{K_{fa}+{\left[Tot\right]}_{fa}^{II}}\right)$$

for the facilitated transporter (fa). [*A*]^*I*^ is the concentration (mol/l) of *substrate A* in *compartment I* and [Tot]^*I*^ is the total sum of all substrates of the exchanger or the facilitative transporter in *compartment I*, while [R]_ex_^*I*^ is the sum of all exchanger substrates, excluding *substrate A*. *K* is the dissociation constant (mol/l) for the exchanger (*K*_ex_) or facilitated transporter (*K*_fa_), and the maximum transport rate (*V*_max_, mol/min) is *V*_ex_ for the exchanger or *V*_fa_ for the facilitated transporter.

Intracellular amino acids were represented by two generic amino acids, to differentiate between substrates of the transporters that transport phenylalanine by both exchange and facilitated transporters and substrates transported by exchange transporters only. For the first generic amino acid, i.e., substrates transported by facilitated transporters [SLC16A10 (TAT1), SLC43A1 (LAT3), and SLC43A2 (LAT4)] and exchangers [SLC7A5 (LAT1) and SCL7A8 (LAT2)], which includes phenylalanine (alanine, isoleucine, leucine, methionine, phenylalanine, tyrosine, tryptophan, and valine), the sum of the intracellular concentrations available in the literature for these amino acids, 3,132 μmol/l, was applied (19, 24). For the second generic amino acid, the sum of those intracellular concentrations available in the literature for amino acids transported by exchangers (but not facilitated transporters), including phenylalanine (asparagine, cysteine, glutamine, glycine, histidine, serine, and threonine), 4,491 μmol/l, was applied. Previous data indicate few instances of significant decline in intracellular amino acid concentrations in perfused human placentas provided with glutamate over the course of an experiment (11). As such, the intracellular concentrations of the two generic amino acids within the model were kept constant throughout the experiment.

MVM and BM exchangers were assumed to be symmetrical, with the same dissociation constants on either side of the membrane in MVM and BM (*K*_ex_ = 200 μmol/l and *K*_fa_ = 1,000 μmol/l) (22). At steady state, net transfer in the model will be the same whether or not transporters are symmetrical. While it remains to be established how this affects the system for different operating conditions, we did not want to introduce additional model parameters without experimental justification. *V*_max_ values were fitted by manual adjustment of the parameters, so that the model matched the average of the experimental steady-state placental uptake and transfer values over all flow conditions. In the first instance, the BM exchanger and facilitated transporter were assumed to have the same *V*_max_ to reduce the number of parameters required for the model. For uptake under physiological amino acid concentrations, we represented maternal input amino acid concentrations with the approaches of the two generic amino acids described above using literature values (19). With maternal values for the facilitated substrates, this value was 615 μmol/l, and for the exchanger-only substrates, this value was 915 μmol/l.

#### Flow modeling.

Blood flow into and out of the maternal and fetal compartments results in a net molecular flux *J*_*A*,flow_^*i*^ (mol/min) as follows

$$J_{A,flow}^{i}=F_{i}\left({\left[A\right]}_{in}^{i}-{\left[A\right]}^{i}\right)$$

where [*A*]_in_^*i*^ is the inlet concentration (mol/l) of *substrate A* in *compartment i* and [*A*]^*i*^ is the concentration of *substrate A* in *compartment i*. F_*i*_ is the constant flow rate into and out of *compartment i* (l/min).

#### Metabolic modeling.

Metabolism of amino acids was represented by linear kinetics with the assumption of an unsaturated process with rate constant *k*_metab_. The rate constant was determined simultaneously by fitting average steady-state amino acid uptake and transfer

$$J_{A,metab}^{S}=K_{metab}{\left[A\right]}^{s}$$

where *J*_*A*,metab_^s^ is the metabolic rate (mol/min), [*A*]^s^ is the concentration (mol/l) of *substrate A* in the syncytiotrophoblast, and *k*_metab_ is the rate constant (l/min). This equation represents all metabolic removal of phenylalanine, and as there is no phenylalanine hydroxylase activity, this equation is likely to represent primarily protein synthesis incorporation. The release of amino acids from the protein pool was not modeled, as median protein half-lives are considerably longer than the course of this experiment (25).

#### Diffusion modeling.

To determine if diffusion could explain the transfer of phenylalanine, the effective diffusive permeability was fitted by manual adjustment of this parameter, so that the model matched the experimental average steady-state placental uptake. The following equation was used to model the flux due to simple diffusion

$$J_{A,dif}^{m\to f}=V_{dif}\left({\left[A\right]}^{m}-{\left[A\right]}^{f}\right)$$

where *V*_dif_ is the effective diffusive permeability constant (l/min).

#### Compartmental modeling.

A compartmental modeling approach was adopted on the basis of our previous work (30), in which the placenta was represented as three separate volumes corresponding to the maternal intervillous space, syncytiotrophoblast, and fetal capillaries. All compartments were assumed to be well mixed. The transfer of amino acids between the compartments was modeled as fluxes mediated by the exchange transporters at the MVM and facilitative and exchange transporters at the BM

$$\frac{d{\left[A\right]}^{m}}{dt}=\frac{1}{v_{m}}\left(J_{A,flow}^{m}-J_{A,ex}^{m\to s}-J_{A,dif}^{m\to f}\right)\;\;\left(\text{intervillous space}\right)$$

$$\frac{d{\left[A\right]}^{s}}{dt}=\frac{1}{v_{s}}\left(J_{A,ex}^{m\to s}-J_{A,ex}^{s\to f}-J_{A,fa}^{s\to f}-J_{A,metab}^{s}\right)\;\left(\text{syncytiotrophoblast}\right)$$

$$\frac{d{\left[A\right]}^{f}}{dt}=\frac{1}{v_{f}}\left(J_{A,flow}^{f}+J_{A,ex}^{s\to f}+J_{A,fa}^{s\to f}+J_{A,dif}^{m\to f}\right)\;\;\left(\text{fetal capillary}\right)$$

where [*A*]^*i*^ is the concentration (mol/l) of *substrate A* in *compartment i* and v_*i*_ is the volume of *compartment i* (liters). *J*_*A,x*_^*i→j*^ represents the net molecular flux (mol/min) of *substrate A* from *compartment i* to *compartment j* mediated by transporter *x*. Here m, s, and f represent the maternal, syncytiotrophoblast, and fetal compartments, respectively, while ac, ex, and fa denote the accumulative, exchange, and facilitative transporters, respectively. *J*_*A*,flow_^*i*^ is the net molecular flux of *substrate A* due to blood flow, *J*_*A*,metab_^s^ is the consumption of *substrate A* by metabolic processes (for phenylalanine, primarily protein synthesis), and *J*_*A*,dif_^m→f^ is flux due to paracellular diffusion. Model equations were implemented in MATLAB (R2014b). Simulations were carried out for transporters alone or for diffusive transfer alone by omission of the relevant terms in the compartmental model equations.

Parameter variation was undertaken in the final transport model to mimic metabolism by modeling the effect of a fivefold increase and decrease in model parameters on uptake or transfer of phenylalanine.

### Statistics

Data were analyzed by two-way ANOVA, with maternal and fetal flow as discrete variables. Linear regression analysis was performed to compare experimental data with model predictions. Values are means ± SE; *n* is the number of placentas.

## RESULTS

### 

#### Perfusion data.

The average cotyledon weight was 42.0 ± 9.7 g (*n* = 5 placentas) and maternal flow rates were 10, 14, and 18 ml/min; these values equate to maternal flow rates of 0.31 ± 0.08, 0.43 ± 0.11, and 0.55 ± 0.14 ml/g placental cotyledon. For fetal flow rates of 3, 6, and 9 ml/min, these values equate to fetal flow rates of 0.09 ± 0.02, 0.18 ± 0.05, and 0.28 ± 0.07 ml/g placental cotyledon. Average fetal perfusate recovery was 5.88 ± 0.06 ml/min at the beginning and 5.76 ± 0.09 ml/min at the end (at 6 ml/min flow rate) of the experiment. Average maternal recovery was 13.92 ± 0.08 ml/min at the beginning and 13.92 ± 0.08 ml/min at the end (at 14 ml/min flow rate) of the experiment.

#### Placental uptake of [^14^C]phenylalanine.

**Fig. 2.**
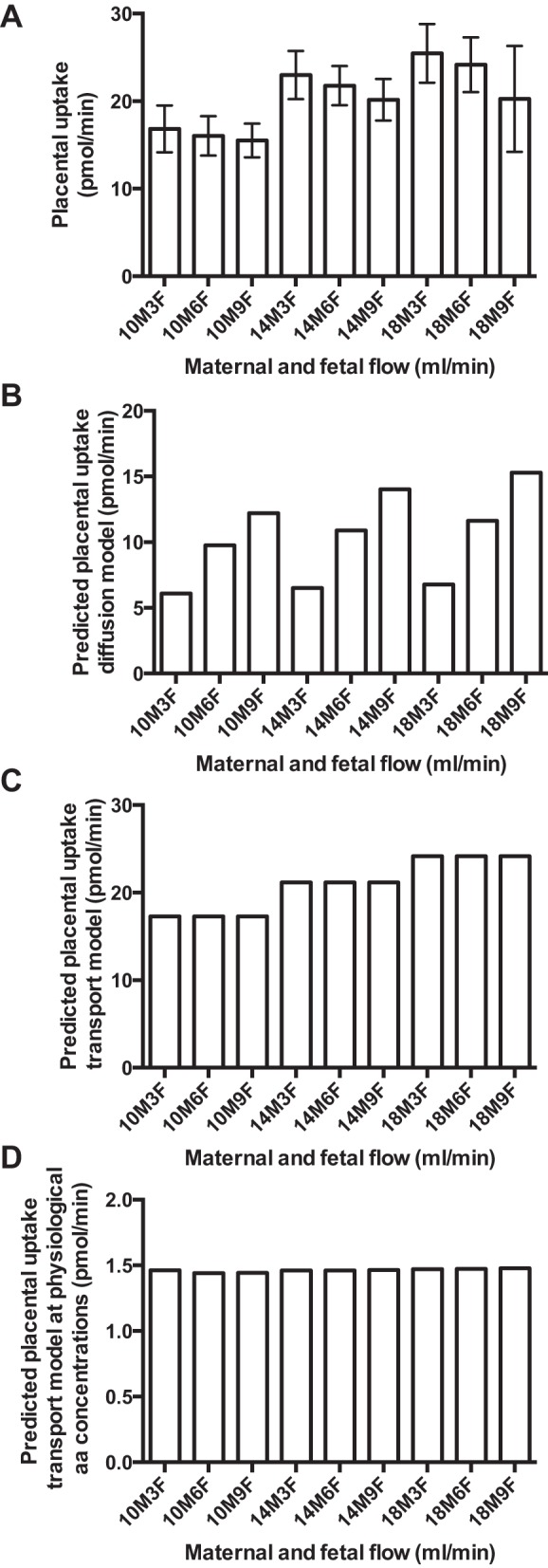
Placental phenylalanine uptake from maternal circulation: experimental data and predicted transfer under certain assumptions. 10M, 14M, and 18M, maternal flow of 10, 14, and 18 ml/min; 3F, 6F, and 9F, fetal flow of 3, 6, and 9 ml/min. *A*: experimental uptake of [^14^C]phenylalanine across the perfused placental lobule. Uptake of [^14^C]phenylalanine from the maternal circulation was associated with maternal (*P* = 0.011), but not fetal (*P* = 0.41), flow rates. There were no significant interactions between maternal and fetal flow (*P* = 0.96). Values are means ± SE; *n* = 5 placentas. *B*: predicted uptake of phenylalanine if transfer is mediated by simple diffusion. Maternal uptake levels could not be matched, and there was no correlation between predicted uptake and experimental data (*R*^2^ = 0.02). *C*: predicted uptake of phenylalanine if transfer is mediated by facilitated and exchange transporters. There was good correlation between predicted uptake and experimental data (*R*^2^ = 0.77). *D*: predicted uptake of phenylalanine tracer at physiological maternal arterial amino acid levels if transfer is mediated by transporters and with the assumption of intracellular metabolism or compartmentalization. Because amino acid (aa) concentrations within the perfusate are much higher, delivery is no longer rate-limiting and maternal flow does not determine uptake.

**Fig. 3.**
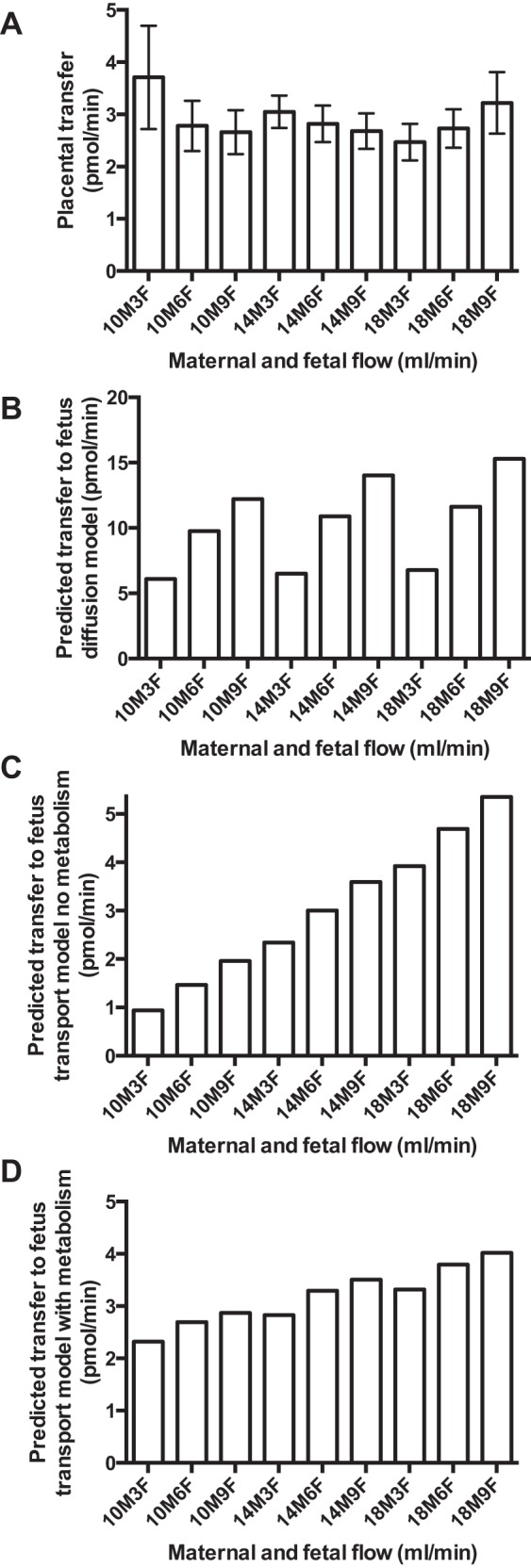
Placental phenylalanine transfer: experimental data and predicted transfer under certain assumptions. *A*: experimental transfer of [^14^C]phenylalanine across the perfused placental lobule. Transfer of phenylalanine to the fetal circulation was not related to maternal (*P* = 0.89) or fetal (*P* = 0.94) flow rates, and there were no interactions between maternal and fetal flow (*P* = 0.95). Values are means ± SE; *n* = 5 placentas. *B*: predicted transfer of phenylalanine if transfer is mediated by simple diffusion. Uptake and transfer are equal, and fetal flow has the predominant effect on transfer. *C*: predicted transfer of phenylalanine if transfer is mediated by transporters and with the assumption of no intracellular metabolism or compartmentalization. Because uptake is greater than transfer, intracellular phenylalanine concentrations rise over time, driving a progressive increase in transfer over the course of the experiment. This scenario does not reflect the experimental data. *D*: predicted transfer of phenylalanine if transfer is mediated by transporters and with the assumption of intracellular metabolism or compartmentalization. While transport with metabolism demonstrates the closest agreement with the experimental data (*R*^2^ = 0.14), none of the model outputs showed good correlation, indicating that other factors are required to fully account for the mechanisms underlying transfer of phenylalanine.

Placental uptake of [^14^C]phenylalanine increased with increasing maternal flow (*n* = 5 placentas, *P* = 0.011) but was not related to fetal flow (Fig. 2*A*). There were no significant interactions between maternal and fetal flow. The computational model was used to predict placental uptake, with the assumption of simple diffusion (Fig. 2*B*) and transport (MVM exchange and BM-facilitated transport and exchange; Fig. 2*C*). The experimental data were most consistent with uptake by transport (*R*^2^ = 0.77), rather than simple diffusion (*R*^2^ = 0.02). The simulation was also conducted in the presence of physiological maternal concentrations of amino acids. In this case, there was only a marginal effect of flow on phenylalanine uptake (Fig. 2*D*). Predicted uptake was essentially identical if modeled with or without placental metabolism; therefore, in Fig. 2, *C* and *D*, the predictions show uptake for the model that included metabolism, to match Fig. 3*D*.

#### Placental transfer of [^14^C]phenylalanine.

Net flux of [^14^C]phenylalanine (mol/min) to the fetus was unaffected by varying fetal (*P* = 0.89) or maternal (*P* = 0.94) flow rates, nor was there an interaction between maternal and fetal flow (*P* = 0.95, *n* = 5; Fig. 3*A*). As such, the increase in placental uptake with increasing maternal flow did not translate into an increased transfer to the fetal circulation. The computational model was used to predict placental transfer with the assumption of simple diffusion (Fig. 3*B*) and transport (MVM exchange and BM-facilitated transport and exchange), first, in the absence of metabolism or compartmentalization (Fig. 3*C*) and, second, with the assumption of metabolism and/or compartmentalization (Fig. 3*D*). The second model, with the assumption of syncytiotrophoblast metabolism and/or compartmentalization, provided the best overall representation of the experimental data, as observed in Fig. 3*D*. Nonetheless, there was still a progressive increase in phenylalanine transfer associated with increasing maternal flow, although the fit was less convincing than that for uptake (*R*^2^ = 0.12). The model could be effectively fitted to the experimental data only if intracellular phenylalanine concentration was kept constant within the syncytiotrophoblast.

#### Baseline uptake and transfer over time.

[^14^C]phenylalanine uptake and transfer were compared at baseline maternal and fetal flow rates (14 and 6 ml/min, respectively) at the beginning, middle, and end of the experiment. At baseline flow rates, there were no differences in maternal venous concentration (mol/l) or placental uptake (mol/min) over the course of the experiment.

#### Creatinine transfer.

**Fig. 4.**
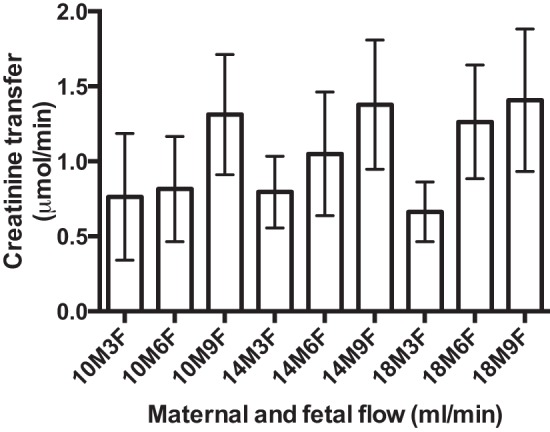
Creatinine transfer across the perfused human placenta. Creatinine transfer was not significantly related to maternal flow rate (*P* = 0.84), but there was a significant relationship with fetal flow rate (*P* = 0.015). There was no interaction between maternal and fetal flows (*P* = 0.94). Values are means ± SE; *n* = 5 placentas.

Creatinine transfer was significantly related to fetal (*P* = 0.015), but not maternal, flow rate, and there was no interaction between fetal and maternal flow rate (*n* = 5 placentas; Fig. 4).

#### Sensitivity analysis for the transport model, including metabolism.

**Fig. 5.**
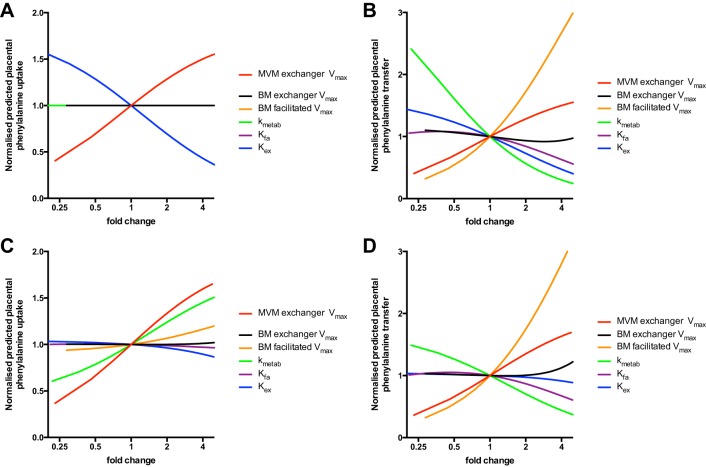
Parameter variation showing the predicted effect of a 5-fold increase and decrease, respectively, in model parameters on uptake and transfer of phenylalanine for the experimental paradigm (*A* and *B*) and modeled with physiological amino acid concentration (*C* and *D*). Lines for basal plasma membrane (BM)-facilitated *V*_max_ and *K*_fa_ are obscured by BM exchanger *V*_max_. *A*: sensitivity analysis for placental uptake indicates that placental uptake is dependent on the ratio of *K* to *V*_max_ in the linear transport regimen. *B*: sensitivity analysis for placental transfer indicates that placental transfer is highly sensitive to metabolic rate and *V*_max_ of the BM-facilitated transporter. This sensitivity analysis is based on the low uterine arterial phenylalanine concentration used in the experimental model. Parameter variation shows the predicted effect of a 5-fold increase and decrease, respectively, in model parameter on uptake or transfer of phenylalanine under conditions assumed for physiological modeling. *C*: sensitivity analysis for placental uptake under conditions assumed for physiological modeling indicates that placental uptake is dependent on MVM exchanger *V*_max_ and metabolic rate. There is a marked difference between predicted sensitivities under experimental and physiological conditions. *D*: sensitivity analysis for placental transfer under conditions assumed for physiological modeling indicates that placental transfer is sensitive to metabolic rate and *V*_max_ of the MVM and BM-facilitated transporters.

Parameter variation was undertaken in the transport model with metabolism included, in which the effect of a fivefold increase and decrease in model parameter on the uptake/transfer of phenylalanine was considered. The sensitivity analysis for uptake indicated that *V*_max_ and *K* for the exchanger on the MVM are the major determinants of transfer (Fig. 5). The sensitivity analysis for transfer indicated that the *V*_max_ of the facilitated transporter on the BM and then the *V*_max_ of the exchanger on the MVM are major determinants of transfer when metabolic rate is applied as a major limiting factor. Sensitivity analysis showed that uptake under experimental conditions was dependent on the ratio of *K* to *V*_max_ in the linear regimen (Fig. 5*A*). In the case of the BM, transfer was highly sensitive to the rate of metabolism and *V*_max_ of the facilitated transporter (Fig. 5*B*). However, when sensitivity analysis, including physiological amino acid concentrations, was performed, distinct differences were observed, particularly in regard to uptake, where metabolism and, to a lesser degree, facilitated transporter *V*_max_ now affected the model (Fig. 5, *C* and *D*).

#### Tissue and protein counts and mass balance.

On the basis of the steady-state measurements over the course of the experiment, [^14^C]phenylalanine uptake per cotyledon was 4.6 ± 0.7 nmol, 15%, 0.7 ± 0.02 nmol, of which was transferred to the fetal circulation, leaving 3.9 ± 0.01 nmol retained within the perfused cotyledon (*n* = 5 placentas). After protein precipitation, ^14^C label was measured in the supernatant and in the protein pellet derived from the perfused cotyledon. The concentration of free [^14^C]phenylalanine in the tissue was 1.0 ± 0.7 nmol/cotyledon and the amount of [^14^C]phenylalanine incorporated into protein was 1.2 ± 0.5 nmol/cotyledon. Total recovery of [^14^C]phenylalanine was 2.2 ± 0.8 nmol/cotyledon, which equates to 56% of the tracer retained in the tissue (*n* = 5 placentas).

#### Estimation of [^14^C]phenylalanine gradient across the BM.

On the basis of a tissue [^14^C]phenylalanine content of 1.03 nmol/cotyledon, with a mean wet weight of 42 g, and the assumption that the trophoblast occupies 15% of placental volume (21), the placental [^14^C]phenylalanine concentration was calculated to be ∼163 nmol/l vs. fetal vein concentrations of 1 nmol/l at low flow rates and 0.3 nmol/l at high flow rates.

## DISCUSSION

This study demonstrates that factors additional to transporter activity and flow determine placental transfer of phenylalanine to the fetal circulation. Understanding the key determinants of amino acid transfer is essential if we are to identify the underlying causes of impaired amino acid transfer associated with fetal growth restriction and mechanistic targets for potential interventions. The observation that increased placental uptake of phenylalanine did not lead to corresponding increases in transfer to the fetal circulation suggests that other factors, such as incorporation into protein, are potentially rate-limiting. This has important implications for our understanding of the regulation of amino acid transfer in the human placenta.

The experiments performed here allowed investigation of the dependence of placental uptake of phenylalanine from the maternal circulation and transfer to the fetal circulation across intact placental tissue on flow and the application of computational modeling to interpret the transfer mechanisms that underlie these processes. The observation that phenylalanine uptake was limited by maternal flow was consistent with modeling predictions and illustrates that, at the concentration of [^14^C]phenylalanine used in these experiments (2.7 pmol/l), supply was inadequate to saturate transport capacity. However, at physiological concentrations of amino acids (∼40 μmol/l for phenylalanine), supply is unlikely to become limiting; while this needs to be demonstrated experimentally, the model implies that phenylalanine uptake would not be flow-limited at physiological concentrations. The demonstration that the experimental uptake data matched the transport model, rather than the diffusion model, illustrates the role of transporters and confirms that phenylalanine transfer in the perfusion system is occurring by predicted mechanisms. The pattern of uptake could not be fitted by the diffusion model, which showed a strong effect of fetal flow that was not observed in the experimental data.

We initially proposed that phenylalanine transfer could be flow-limited on the BM, with its transfer across the BM of the placental syncytiotrophoblast mediated by facilitated diffusion (8). However, phenylalanine transfer occurred at a near-constant rate across the range of fetal flow rates. This is consistent with a high intracellular concentration within placental tissue relative to the capillary concentration, as, in this case, changes in fetal flow will have a relatively small effect on the overall gradient and transfer. However, the transmembrane gradient should have increased during the experiment both in response to changes in [^14^C]phenylalanine uptake with increasing flow and because uptake exceeded efflux. Although phenylalanine uptake was ∼40% greater at the fastest than at the slowest maternal flow rate, this did not translate into increased transfer to the fetal circulation. Moreover, as illustrated by computational modeling, the observation that phenylalanine uptake was greater than efflux implies that intracellular phenylalanine concentration should have been increasing with time, driving increased transfer to the fetal circulation (Fig. 3*C*). Explanations for this discrepancy between uptake and transfer include the following: *1*) the intracellular concentration of free [^14^C]phenylalanine, available for transfer, is controlled by another factor, such as metabolism, or *2*) the activity of the facilitated transporters on the BM is not primarily determined by the transmembrane concentration gradient.

BM-facilitated transport might not be determined by the transmembrane concentration gradient for the following reasons. First, BM-facilitated transport was saturated; however, this is unlikely, as tracer concentration is well below the *K*_m_, and, in this range, flux should increase proportionally with concentration (even if transfer of the unlabeled substrate were saturated). Second, facilitated amino acid transporters do not operate as we would expect on the basis of observations of other facilitated transporters such as GLUT1 (SLC2A1). Facilitated transport of glucose by GLUT1 (SLC2A1) appears to be dependent on the transplacental concentration gradient, and it seems reasonable to assume that facilitated amino acid transporters may share this characteristic (10, 29). However, the facilitated transporters LAT3 and LAT4 are reported to have complex kinetics with multiple apparent affinities for phenylalanine (3). It is therefore possible that they do not operate in a manner consistent with previous observations for other facilitated transporters. Further characterization of these transporters is therefore warranted to help clarify this issue.

It is noteworthy that up to two-thirds of the [^14^C]phenylalanine retained within the placenta was incorporated into protein. If this phenomenon applies to other amino acids, this would reduce the intracellular concentration of amino acids and, therefore, the amino acid concentration gradient driving transfer to the fetus. Previous work in the guinea pig has suggested an important role for protein metabolism in amino acid transfer (4). Alternatively, phenylalanine catabolism or sequestration within intracellular organelles could regulate the free amino acid pool available for transport. When metabolism was included in the model, the predicted transfer to the fetus was much closer to the observed experimental data. Nevertheless, if we assume linear kinetics for metabolism, the model could not fully reproduce the constant rate of phenylalanine transfer observed experimentally. The reason for this is that, in the model, increased maternal uptake would directly lead to a higher equilibrium of intracellular amino acid concentration, increasing the concentration gradients that drive amino acid transport across the BM. Only if intracellular phenylalanine concentration was fixed could the model provide a good representation of our experimental data. Therefore, for metabolism to fully explain the data, regulation of metabolism would be needed to maintain a constant free intracellular phenylalanine concentration at the BM interface.

A relatively high proportion of ^14^C label was unaccounted for; this has been reported previously, but the cause for this remains elusive (26). Catabolism is a possibility, but as phenylalanine hydrolase is not expressed in the placenta, we consider this unlikely (25). It is possible that phenylalanine uptake may have been overestimated, as our calculations were based on steady-state values, which may not fully reflect equilibration time following changes in flow rate. Another possibility is that the observed quenching in protein extracts was not fully accounted for. In both cases, the proportion of [^14^C]phenylalanine incorporated into protein would have been underestimated, affecting estimates of tracer recovery.

The observation that much of the [^14^C]phenylalanine taken up was incorporated into protein suggests that metabolism and integration of phenylalanine into protein make a significant contribution. However, whether metabolism can fully explain the discrepancy between the model and experimental data or whether there is a combination of metabolism and some other factor, such as compartmentalization or facilitated transporter function, remains to be determined. For example, compartmentalization of the amino acid arginine has been proposed as an explanation of the arginine paradox in nitric oxide production (13). Protein synthesis inhibitors have been shown to be effective in inhibition of protein synthesis in the perfused placenta, and it would be interesting to determine if protein synthesis inhibitors also stimulated the transfer of amino acids to the fetus (2).

While all the factors modeled are necessary for transfer, it is important to identify those that are most likely to become rate-limiting and, thus, have the greatest clinical relevance. The model sensitivity analysis identifies those factors that, if we assume that the model is correct, have the greatest impact on phenylalanine transfer. It is important to note that the sensitivity analysis favored different factors under experimental (low phenylalanine concentrations) and physiological amino acid concentrations. However, it appears that MVM exchanger activity, BM-facilitated transporter activity, and incorporation of amino acids into protein were predicted to be the primary determinants of placental transfer.

As the experiments themselves were not performed with physiological concentrations of amino acids, we should be careful about extrapolating our findings to the physiological situation. However, while experimental validation is required, we are confident that the modeling framework is capable of effectively representing the main transport processes relevant for physiological fluids such as serum. We should also note that the placenta is a more complex tissue than the model currently reflects and that processes such as metabolism may occur in cell types other than the syncytiotrophoblast.

In the normal placenta, maternal and fetal flow rates are on the order of 2 and 0.2 ml·g placenta^−1^·min^−1^, respectively (17). In this study our maternal flow rates were below normal (15–30% of physiological), while our fetal flow rates spanned the normal range (50–140% of physiological). Maternal blood flows in this range or fetal flow of 50% would normally be associated with placental disease, leading to preeclampsia or fetal growth restriction (16).

This model is based on human full-term placenta but could be applied to other gestational ages and species. By substitution of the data on the volumes of the different tissue compartments, rates of uterine and umbilical blood flow, and the localization of transporters, the basic model would be applicable to a range of species or gestational ages.

Creatinine transfer across the human placenta is generally believed to occur via paracellular diffusion (31), and, consistent with this notion, the experimental data followed the pattern predicted by the diffusion model, providing confidence in our modeling approaches.

The perfusion system provides an excellent model for study of placental transfer (27). Nevertheless, there are issues that should be considered when the data are interpreted. First, maternal-side perfusion may not fully represent the uteroplacental perfusion that occurs in vivo via the spiral arteries (26). This may affect the efficiency of mixing within the intervillous space and, thus, the efficiency of transfer. Second, this study measured the transfer of one amino acid in the absence of other amino acids that would normally be present. Amino acid transfer is likely to be determined by interaction between amino acids, and future studies including all amino acids would be informative (18).

In considering these observations, we also need to be mindful of the time course of these experiments, as the factors limiting transfer over the course of 3 h may be different from those limiting transfer over days, weeks, and longer. While incorporation of phenylalanine into the protein pool and metabolism may predominate over short time frames, in the longer term, transport may affect amino acid availability for protein synthesis, the size of the protein pool, and, thus, transfer over extended periods. It is likely that protein synthesis and breakdown are in quasi-steady state, with input and output matched over time. This would allow the placenta to maintain supply to the fetus in response to short-term variations in maternal supply. If a significant proportion of placental amino acids enters a protein pool before being transported to the fetus, we may need to rethink the time frames over which amino acid transfer is regulated.

In fetal growth-restricted pregnancies, many placental factors have been shown to be altered; some of these will be key determinants of placental function, while others will not. A key aim of the model is to be able to identify the factors that are most likely to be rate-determining for placental transfer. These factors are the most likely to become rate-limiting in fetal growth-restricted pregnancies and need to be targeted for successful interventions. By modeling the phenotypes observed in fetal growth restriction, we hope to identify the factors that are having the greatest effect on placental function and, thus, fetal growth, as these are the most important targets for future research.

In conclusion, this study suggests that transporter activity is a major determinant of phenylalanine transfer across the perfused human placenta, but flow is not. However, our combined experimental and computational modeling approach leads us to conclude that other factors, such as metabolism and integration into protein within the placenta, play a previously underappreciated role.

## GRANTS

This work was funded by Biotechnology and Biological Sciences Research Council Project Grants BB/I011250/1 and BB/I011315/1.

## DISCLOSURES

No conflicts of interest, financial or otherwise, are declared by the authors.

## AUTHOR CONTRIBUTIONS

E.M.L. and S.B. performed the experiments; E.M.L., S.P., S.B., N.P., B.G.S., and R.M.L. analyzed the data; E.M.L., S.P., N.P., B.G.S., and R.M.L. interpreted the results of the experiments; E.M.L., S.P., B.G.S., and R.M.L. prepared the figures; E.M.L., B.G.S., and R.M.L. drafted the manuscript; E.M.L., S.P., S.B., I.P.C., J.D.G., E.D.J., N.P., C.P.S., K.L.W., B.G.S., and R.M.L. edited and revised the manuscript; E.M.L., S.P., S.B., I.P.C., J.D.G., E.D.J., N.P., C.P.S., K.L.W., B.G.S., and R.M.L. approved the final version of the manuscript; S.B., I.P.C., J.D.G., E.D.J., N.P., C.P.S., K.L.W., B.G.S., and R.M.L. developed the concept and designed the research.
